# A Novel Immunotype-based Risk Stratification Model Predicts Postoperative Prognosis and Adjuvant TACE Benefit in Chinese Patients with Hepatocellular Carcinoma

**DOI:** 10.7150/jca.54408

**Published:** 2021-03-15

**Authors:** Tian-En Li, Ze Zhang, Yi Wang, Da Xu, Jian Dong, Ying Zhu, Zheng Wang

**Affiliations:** 1 Department of General Surgery, Comprehensive Breast Health Center, Ruijin Hospital, Shanghai Jiao Tong University School of Medicine, Shanghai 200025, China.; 2 Department of General Surgery, Qilu Hospital, Shandong University, Jinan 250012, China.; 3 Department of General Surgery, Huashan Hospital & Cancer Metastasis Institute, Fudan University, Shanghai 200040, China.; 4 Institute of Advanced Surgical Technology and Engineering, First Affiliated Hospital of Medical College, Xi'an Jiaotong University, Xi'an 710061, China.

**Keywords:** Hepatocellular carcinoma, adjuvant TACE, tumor microenvironment, immunotype, prognosis.

## Abstract

**Background and Aims**: The tumor microenvironment can be divided into inflamed, immune-excluded and immune**-**desert phenotypes according to CD8^+^ T cell categories with differential programmed cell death protein 1 (PD-L1) expression. The study aims to construct a novel immunotype-based risk stratification model to predict postsurgical survival and adjuvant trans-arterial chemoembolization (TACE) response in patients with hepatocellular carcinoma (HCC).

**Methods:** A total of 220 eligible HCC patients participated in this study. CD8**^+^** T cell infiltration and PD-L1 expression mode were estimated by immunohistochemical staining. A risk stratification model was developed and virtualized by a nomogram that integrated these independent prognostic factors. The postoperative prognosis and adjuvant TACE benefits were evaluated with a novel immunotype-based risk stratification model.

**Results:** A total of 220 patients were finally identified. Immune-desert, immune-excluded, and inflamed immunotypes represented 45%, 24%, and 31% of HCC, respectively. Univariate and multivariate analyses identified immunotype and PD-L1 expression mode as independent prognostic factors for overall survival time (OS) and recurrence-free survival time (RFS). The nomogram was constructed by integrating immunotype, PD-L1 expression, Barcelona Clinic Liver Cancer (BCLC) stage and tumor grade. The C-index was 0.794 in the training cohort and 0.813 in the validation cohort. A risk stratification system was constructed based on the nomogram classifying HCC patients into 3 risk groups. The average OS times in the low-risk, intermediate-risk and high-risk groups in all cohorts were 77.1 months (95% CI 71.4-82.9), 53.7 months (95% CI 48.2-59.2), and 25.6 months (95% CI 21.4-29.7), respectively. Further analysis showed that OS was significantly improved by adjuvant TACE in the low- and intermediate-risk groups (*P=*0.041 and *P*=0.010, respectively) but not in the high-risk group (*P*=0.398).

**Conclusion:** A novel immunotype-based risk stratification model was built to predict postoperative prognosis and adjuvant TACE benefit in HCC patients. These tools can assist in building a more customized method of HCC treatment.

## Introduction

As the fifth most prevalent cancer and the third most lethal cancer, HCC causes 700,000 deaths each year 1-4. Moreover, HCC is more prevalent in East Asia, Southeast Asia and sub-Saharan Africa 4. Despite the development of surgical techniques, HCC still shows a poor prognosis, mainly attributable to the high rate of metastasis and relapse after curative treatments 5, 6. Therefore, the prevention of relapse is one of the toughest challenges to improve surgical efficacy. Currently, TACE is a major adjuvant management to prevent relapse of HCC and prolong the survival time of patients after hepatectomy 7. Although some clinical studies have reported that HCC patients with large tumors, portal vein tumor thrombus or microvascular invasion benefit from TACE, the role of postoperative adjuvant TACE has not yet been fully established 8, 9. In particular, controversial results reported by several previous studies suggest the need to set a more specific subtype classification and markers to select suitable patients for TACE in addition to the traditional clinical risk factors for recurrence 10, 11.

Recent studies have identified the adaptive or exhausted immune-specific subtypes of HCC on the basis of molecular features 12. However, limited evidence was provided in evaluating prognosis or chemotherapy-related benefits. Multitudinous prognostic signatures have been developed to stratify patients with HCC 13, 14; however, they are mainly concentrated on malignant cells and do not represent stromal cells and the immune microenvironment. Previous studies have shown that the immune response plays a key role in chemotherapy-induced cytotoxicity, which may indicate the patients' response to chemotherapy 15-19. In muscle-invasive bladder cancer, the stromal immunotype has been demonstrated to predict postsurgical survival and adjuvant platinum-based chemotherapy after surgery^15^. Anticancer immunity can be segregated into three main phenotypes: immune-desert, immune-excluded and inflamed phenotypes 20-22. More importantly, collective clinical studies suggest that checkpoint immunotherapy is the most effective in inflamed tumors, as characterized by a high density of IFN-γ-producing CD8**^+^** T cells and the expression of PD-L1 in tumors 23-26. However, the prognostic value and precise function of these three immunotypes in HCC are not quite clear and need to be further investigated.

Therefore, in the present study, we classified HCC patients into three distinctive subtypes of immune desert, immune excluded, or inflamed immunotype and into two PD-L1 expressed modes, and we verified that immunotype and PD-L1 expression were independent prognostic factors. Integrating immunotype, PD-L1 expression and other clinicopathologic features, we constructed a novel risk stratification model, aiming to predict the impact of immunotype on postoperative survival and the benefits of adjuvant TACE therapy. These results may unravel the important role of immune status in HCC and provide a predictive system that could possibly evaluate outcomes for patients who received adjuvant TACE.

## Materials and Methods

### Patients and procedures

This retrospective study was based on clinical data collected from patients diagnosed with HCC for the first time who underwent curative resections at the Department of General Surgery, Huashan Hospital, Fudan University, from January 2012 to December 2015.

The inclusion criteria for patients enrolled in the study were as follows: (1) tumor tissue was histopathologically confirmed as HCC; (2) age >18 and ≤75; and (3) Child-Pugh was classified as A or B by evaluation. Patients were excluded when they met the following criteria: (1) received any preoperative radiotherapy and/or chemotherapy; (2) had recurrent liver cancer or were diagnosed with other cancers; (3) had refractory ascites or severe portal hypertension; and (4) had organ dysfunction. All specimens were pathologically reassessed by two pathologists independently. Human tissues were used with patients' informed consent and the approval of the Clinical Research Ethics Committee of the authors' institution.

A total of 220 HCC patients who underwent curative resections were included in the study. Patients undergoing surgical resection were recommended to undergo adjuvant TACE one or two months after surgery if they encountered any of the following circumstances: having a single tumor with microvascular invasion (MVI), having major vascular invasion, having a single tumor larger than 5 cm or having two or three tumors 27.

TACE was conducted with the Seldinger technique. First, the arterial angiographic catheter was inserted into an appropriate hepatic artery through the femoral artery, and artery angiography was then carried out to show the real artery anatomy. A mixed emulsion of lipiodol, doxorubicin hydrochloride, pirarubicin, or pharmorubicin was slowly infused into the remnant liver by the catheter.

### Specimens and tissue microarray (TMA)

Clinical and follow-up information was analyzed for all patients. OS was calculated from the date of operation to death or the last follow-up. RFS was calculated from the date of resection to the date of tumor recurrence, death or the last follow-up.

Formalin-fixed and paraffin-embedded tumor tissues were employed to build TMAs (in collaboration with Shanghai Biochip Company Ltd, Shanghai, China) as previously described 28. Briefly, to make the TMA, two 4 μm diameter core biopsies from the center and lateral of each donor block were taken. Then, they were transferred to the recipient paraffin block at predefined array positions. In total, 220 pairs of TMA blocks were constructed in this study.

### Immunohistochemistry and evaluation of immunostaining

Immunohistochemical (IHC) staining was carried out as described previously 29. In short, TMAs were deparaffinized and rehydrated, and then the antigen of the tissue was retrieved. Then, the TMAs were incubated with CD8 (BD Pharmingen, 550372, diluted at 1:50) and PD-L1 (Abcam, ab205921, 1:30) primary antibodies at 4°C for 12 hours. After that, the secondary antibody (Dako Denmark A/S, Glostrup, Denmark) was applied and incubated at 37°C for 30 minutes. Diaminobenzidine (DAB) and hematoxylin were used to perform the staining and counterstaining. Two independent pathologists evaluated all specimens.

Three major T cell categories (inflamed, immune excluded, and immune desert) were defined on the basis of the CD8^+^ T cell infiltration pattern at the tumor core and stroma. “Immune desert” was characterized as no significant CD8^+^ T cell infiltration into the tumor core and stroma. CD8^+^ T cell infiltration limited to the invasive margin only was referred to as “immune-excluded”. “Inflamed” immunotype represented diffuse intratumoral CD8^+^ T cell infiltrate. PD-L1^+^ tumor tissues were defined as PD-L1^+^ tumor cells over 5%.

Microvascular invasion (MVI) is a nesting mass of cancer cells observed by microscopy in the lumen of endothelium-lined vasculature, mainly in the branches of portal veins (including intraperitoneal vessels). All the tumor tissues from the HCC patients were pathologically examined by pathologists, and MVI was provided on the pathological report.

### Nomogram construction and validation

A nomogram was constructed based on the data of the multivariable model using R 3.4.3. The 3-year and 5-year OS rates were used to develop a nomogram. C-indexes were calculated to calculate the predictive accuracy. One thousand bootstrap resamples were performed to determine the C-indexes and create calibration plots. Moreover, the patients were divided into three prognostic groups by the risk stratification model depending on each patient's total nomogram score.

### Statistical analysis

All data were expressed as the means ± SEM. Statistical analyses were executed with the aid of SPSS 22.0 (IBM, Armonk, USA) and GraphPad Prism 7 statistical software. Comparisons of categorical and continuous variables were performed with the χ^2^ test or Fisher's exact test. Survival curves were calculated by Kaplan-Meier analysis and compared with the log-rank test. Univariate analysis and Cox regression multivariate analysis were performed to evaluate the independent predictive variables for survival. Differences were defined as significant with two-sided *P*<0.05.

## Results

### Baseline clinicopathological characters

**Figure 1:**
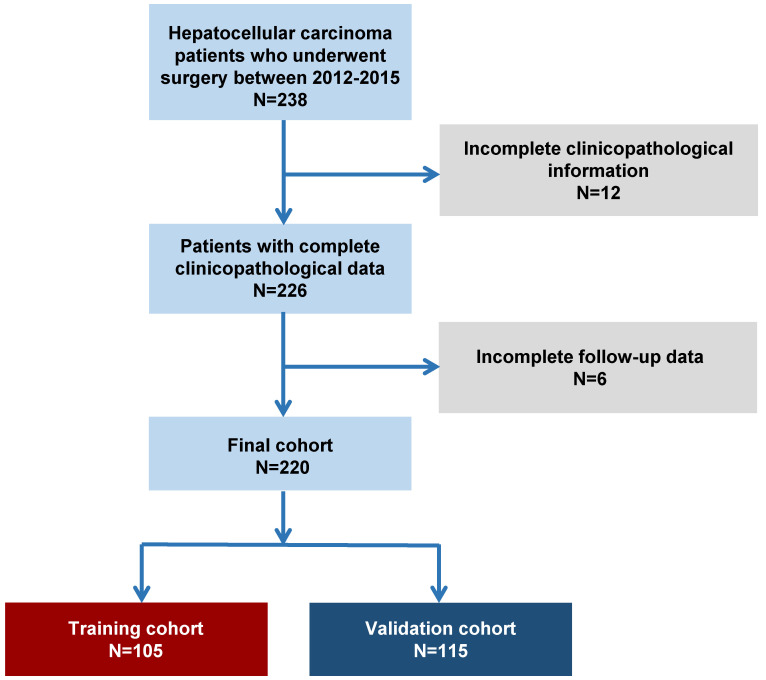
Patient selection flowchart.

The selection of patients was conducted in accordance with the procedure in **Figure 1**. In total, two hundred and twenty patients were included: 105 patients and 115 patients were included in the training and verification cohorts. First, the baseline clinicopathological features of each subgroup were analyzed (**Table 1**). In the training cohort, 89.5% (94/105) were male patients, 68.6% (72/105) of patients were aged ≥50, 92.4% (97/105) were affected with HBV, and 77.1% (81/105) suffered from liver cirrhosis. Patients with AFP ≥20 μg/L accounted for 66.7% (70/105), and those with ALT ≥40 U/L accounted for 54.3% (57/105). In addition, 56.2% (59/105) and 43.8% (46/105) of the patients had Grade 1-2 and Grade 3-4 disease, respectively. In addition, 32.4% (34/105) and 9.5% (10/105) of the patients had HCC with MVI or major vascular invasion, respectively. Moreover, the proportions of patients bearing TNM (AJCC Cancer Staging Manual, Eighth Edition Version) I, II, III and IV tumors were 41.0% (43/105), 33.3% (35/105), 19% (20/105) and 6.7% (7/105), respectively, and the proportions of patients bearing BCLC stage 0-A, B and C tumors were 24.8% (26/105), 30.5% (32/105) and 44.8% (47/105), respectively. Furthermore, 50.5% (53/105) of the patients received TACE therapy postoperatively. Among these 220 patients, 170 patients were recommended adjuvant TACE, and 111 patients received adjuvant TACE after surgical resection.

### Immunotype classification in HCC

**Figure 2:**
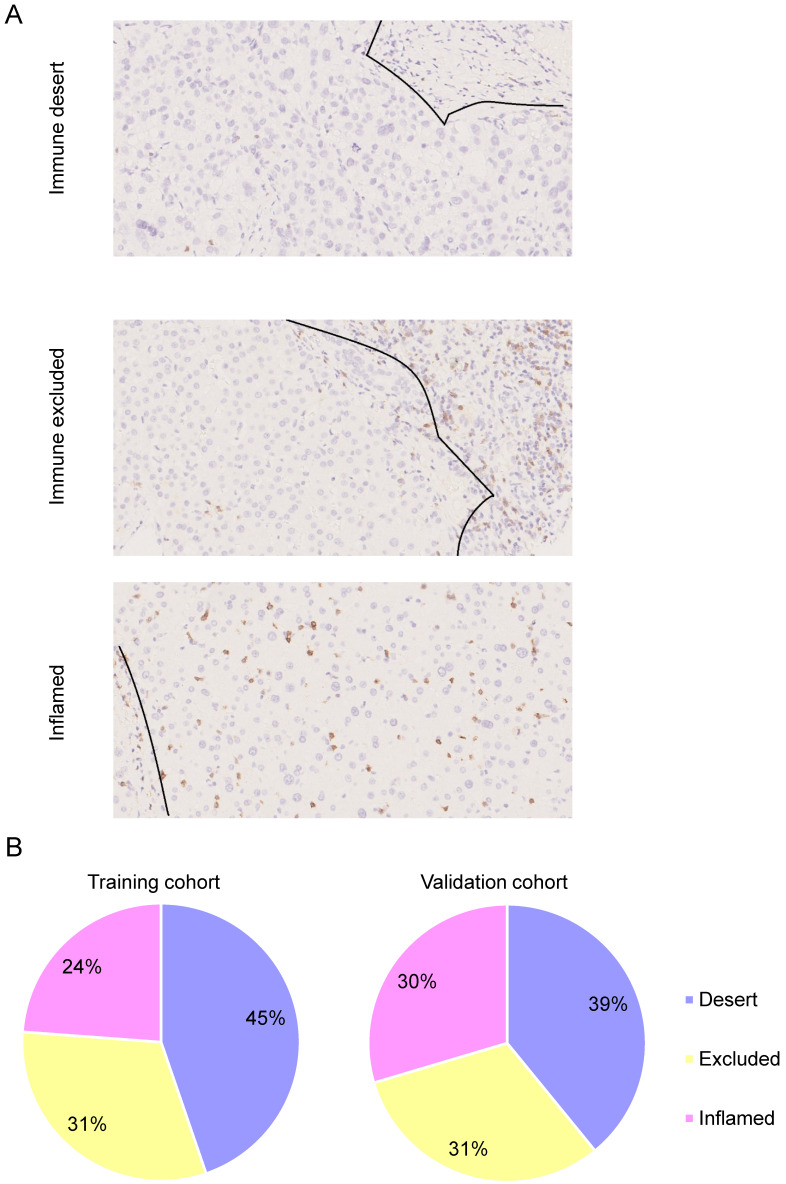
Immunotype classification in HCC. Representative photos of immunohistochemistry staining of CD8^+^ T cells. HCC is characterized as “immune desert” when CD8^+^ T cell infiltration is absent, “immune excluded” when CD8^+^ T cell infiltration is at the invasive margin only, and “inflamed immunotype” when CD8^+^ T cells infiltrate among tumor cells (A). Scale bar: 50 µm. Relative rates of different immunotypes in the training cohort and validation cohort (B).

To assess the categories of CD8^+^ T cell infiltration within HCC and the distribution of intratumoral CD8^+^ T cells in the tumor core and stroma, we performed IHC staining of CD8 in TMAs. Each specimen was categorized on the basis of the distribution of CD8^+^ T cells in relation to the tumor core and stroma. Representative immunohistochemistry images of three major CD8^+^ T cell categories are shown in **Figure 2A**. Among these, 45% had no significant CD8^+^ T cell infiltrate, which was referred to as the “immune desert” (**Figure 2B**). Thirty-one percent had CD8^+^ T cell infiltration limited to the invasive margin only, referred to as “immune-excluded” (**Figure 2B**). Only 24% had diffuse intratumoral CD8^+^ T cell infiltrate, named the “inflamed” immunotype (**Figure 2B**). The relationship between immunotype and clinicopathological parameters is shown in **Table 2**. Immunotype was found to be significantly correlated with HCC grade (*P*=0.024) but not with other clinicopathological features.

**Figure 3:**
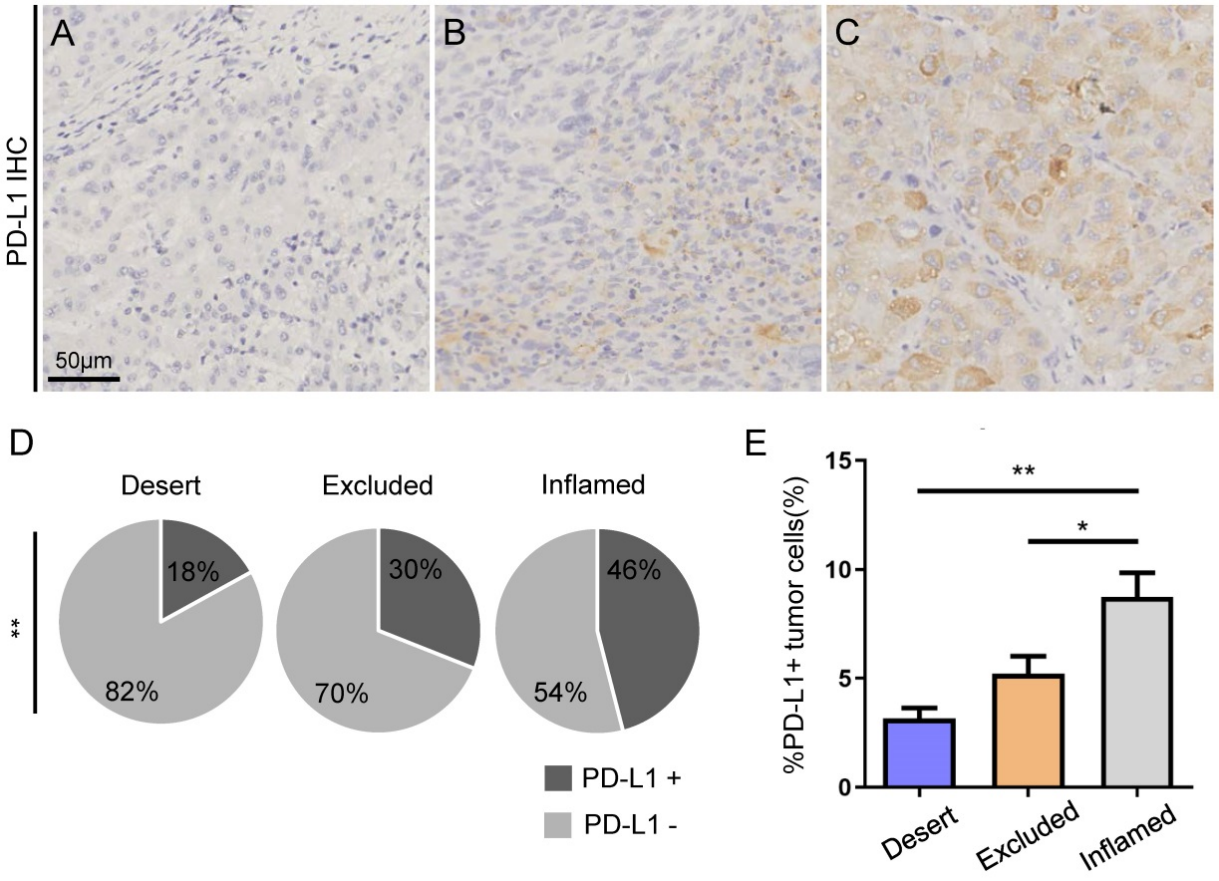
Higher expression of PD-L1 by tumor cells in HCC with inflamed immunotype. Representative photos of immunohistochemistry staining of PD-L1 (A-C). Example of tumors with no expression of PD-L1 (%PD-L1^+^ tumor cells <1%) (A), weak expression (%PD-L1^+^ tumor cells ≥1% and <5%) (B), and strong expression (%PD-L1^+^ tumor cells ≥ 5%) (C). Scale bar: 50 µm. Relative rates of PD-L1^+^ and PD-L1^-^ expression in the patients with different immunotypes in all cohorts (D). PD-L1 expression by tumor cells in HCC with different immunotypes (E).

Moreover, PD-L1 staining by IHC was performed in TMAs. PD-L1^+^ tumor tissues were defined as PD-L1^+^ tumor cells over 5%. Representative immunohistochemistry images of the major PD-L1 expression modes are shown in **Figure 3A-C.** The ratio of PD-L1^+^ tumor cells was found to be significantly higher in HCC patients with inflamed immunotypes than in those with immune-desert and immune-excluded immunotypes (**Figure 3D**). Furthermore, patients with inflamed immunotypes expressed significantly higher PD-L1 than those with immune-desert and immune-excluded immunotypes (**Figure 3E**).

### Impact of immunotype on OS and RFS

**Figure 4:**
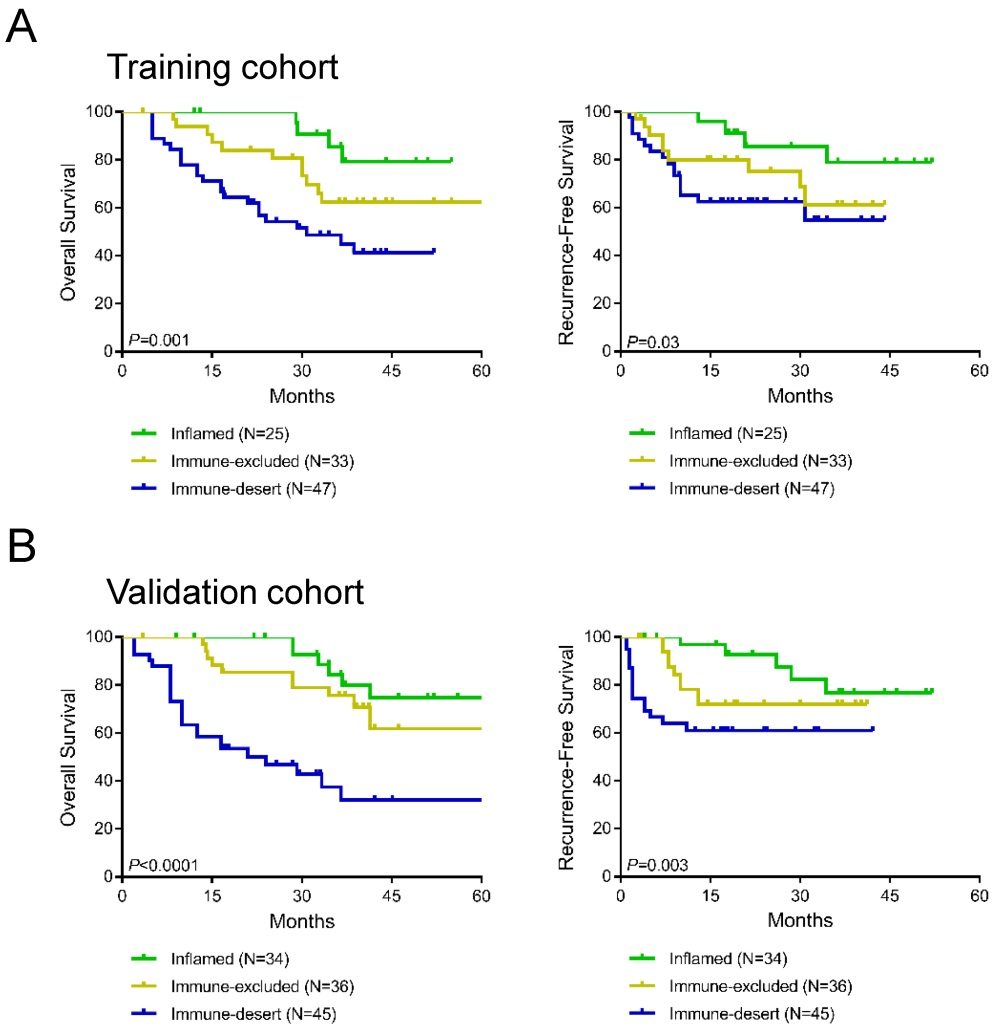
Kaplan-Meier analysis of the impact of immunotypes on prognosis in the training cohort (A) and validation cohort (B).

To further evaluate the prognostic value of immune-desert, immune-excluded, and inflamed immunotypes, Kaplan-Meier analysis was performed to compare OS and RFS between the three immunotype subgroups. In the training cohort, a significant difference was found in OS among all three immunotypes (*P*=0.001, **Figure 4A**), and RFS differed significantly among all three immunotypes (*P*=0.03, **Figure 4A**). Patients with inflamed immunotypes showed the best prognosis and lowest relapse rate, while patients with immune-desert immunotypes showed the poorest prognosis and highest relapse rate. Additionally, the validation cohort showed consistent results with the training cohort (*P*<0.0001 for OS and *P*=0.003 for RFS; **Figure 4B**).

### Univariate and Multivariate Regression Analyses

The Cox hazards model was carried out in the training cohort to exhibit the predictive variables for survival. Univariate analysis showed that clinical factors such as tumor grade (*P*=0.001), BCLC stage (*P*<0.001), immunotype (*P*=0.001) and PD-L1 expression (*P*=0.002) were associated with overall survival time (**Table 3**). Multivariate Cox regression analysis further showed that tumor grade (G 3-4: HR 2.160, 95% CI 1.085-4.292; G 1-2 as reference), BCLC stage (B stage: HR 1.845, 95% CI 1.104-4.237; C stage: HR 3.846, 95% CI 1.304-11.363; 0-A stage as reference), immunotype (immune-excluded immunotype: HR 2.365, 95% CI 1.734-7.616; immune-desert immunotype: HR 4.655, 95% CI 1.600-13.540; inflamed immunotype as reference) and PD-L1 expression (PD-L1 high expression: HR 2.146, 95% CI 1.067-4.310; PD-L1 low expression as reference) were independent predictive variables for OS (**Table 3**).

In addition, univariate analysis and multivariate Cox regression analysis were executed to identify the independent predictive variable for RFS (**Supplementary Table 1**). Tumor grade (G 3-4: HR 4.566, 95% CI 1.832-11.364; G 1-2 as reference), BCLC stage (B stage: HR 5.051, 95% CI 1.621-15.625; C stage: HR 7.692, 95% CI 1.761-33.333; 0-A stage as reference), immunotype (immune-excluded immunotype: HR 3.313, 95% CI 1.133-11.757; immune-desert immunotype: HR 4.330, 95% CI 1.324-14.159; inflamed immunotype as reference) and PD-L1 expression (high expression: HR 6.667, 95% CI 2.660-16.667; low expression as reference) were found to be independent predictive variables for RFS.

Collectively, these results further proved that immunotype and PD-L1 expression were independent prognostic factors for liver cancer. Furthermore, all the independent predictive prognostic factors were included for nomogram construction.

### Nomogram construction and validation

**Figure 5:**
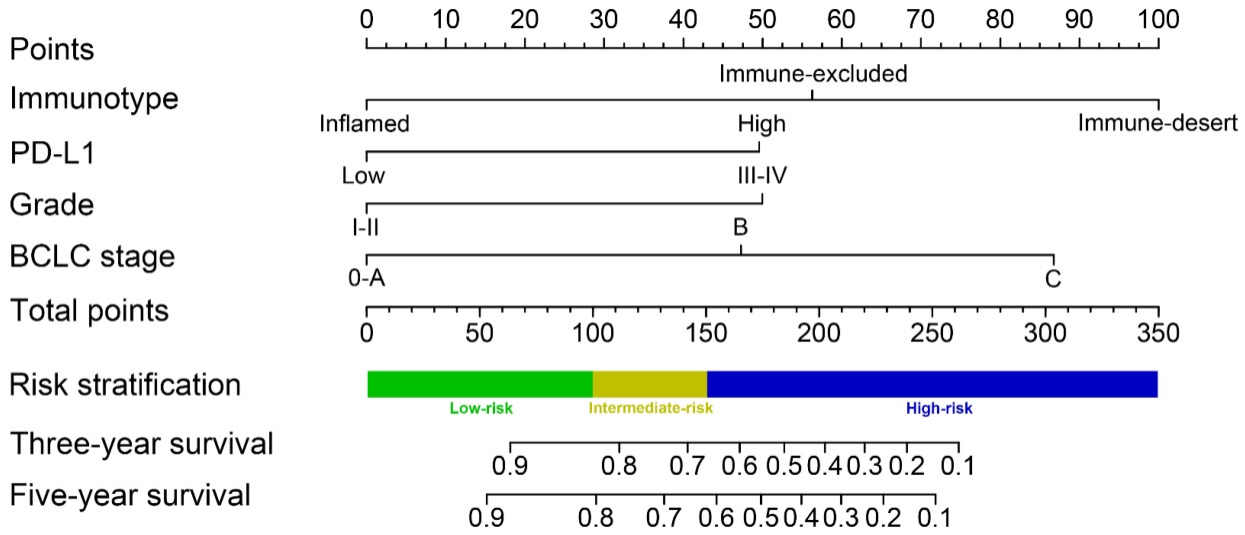
Immunotype-based nomogram for predicting 3- and 5-year overall survival in HCC patients after surgery.

A predictive nomogram was constructed by integrating 3 independent risk factors for survival prognosis (**Figure 5**). Each clinical variable was assigned a score: immunotype (inflamed immunotype scored 0, immune-excluded immunotype scored 56 and immune-desert immunotype scored 100), PD-L1 (PD-L1 low expression scored 0 and PD-L1 high expression scored 50), tumor grade (G 1-2 scored 0 and G 3-4 scored 50) and BCLC stage (0-A stage scored 0, B stage scored 47 and C stage scored 87) (**Table 4**). Furthermore, the total scores were calculated by adding all scores of the included variables. The probability of 3-year and 5-year survival rates were estimated based on the nomogram model.

The C-indexes of the nomogram in the training cohort (0.794, 95% CI 0.732-0.855) and validation cohort (0.813, 95% CI 0.751-0.875) suggested the excellent discrimination ability of the model. In addition, the calibration curves also demonstrated consistency between the predicted and actual 3- and 5-year survival rates in the training cohort (**Supplementary Figure 1A**). The calibration curves of the validation cohort showed good consistency as well (**Supplementary Figure 1B**).

### Risk stratification model development

**Figure 6:**
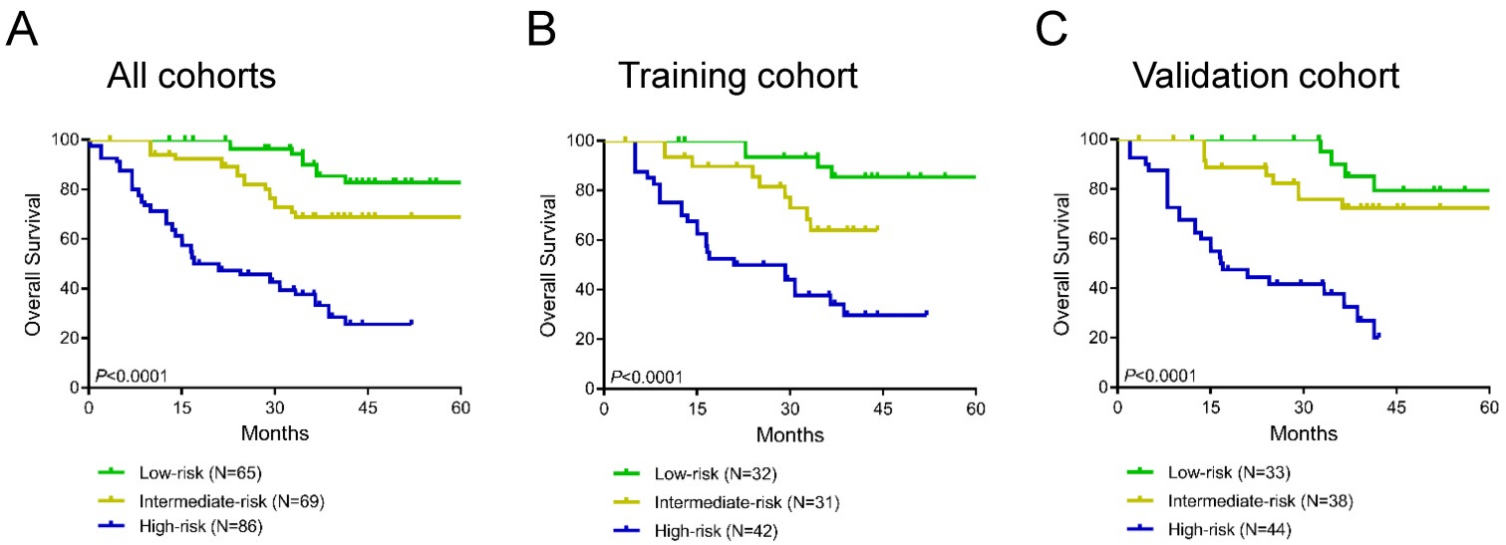
Kaplan curves of low-, intermediate- and high-risk groups in all cohorts (A), the training cohort (B) and the validation cohort (C).

Additionally, we constructed a novel risk stratification model by determining total scores from the nomogram, which separated the patients into three groups with different prognoses. We divided patients into the low-risk group (65/220, total score <100), the intermediate-risk group (69/220, total score 100-150) and the high-risk group (86/220, total score ≥150) on the basis of this model. The Kaplan-Meier survival analysis exhibited the excellent accuracy of this model in predicting prognosis among the 3 risk groups in the training cohort, validation cohort, and all cohorts (**Figure 6A-C**). In all cohorts, the average OS times in the low-risk group, intermediate-risk group and high-risk group were 77.1 months (95% CI 71.4-82.9), 53.7 months (95% CI 48.2-59.2), and 25.6 months (95% CI 21.4-29.7), respectively.

### Survival benefit of adjuvant TACE in groups of stratified risk

**Figure 7:**
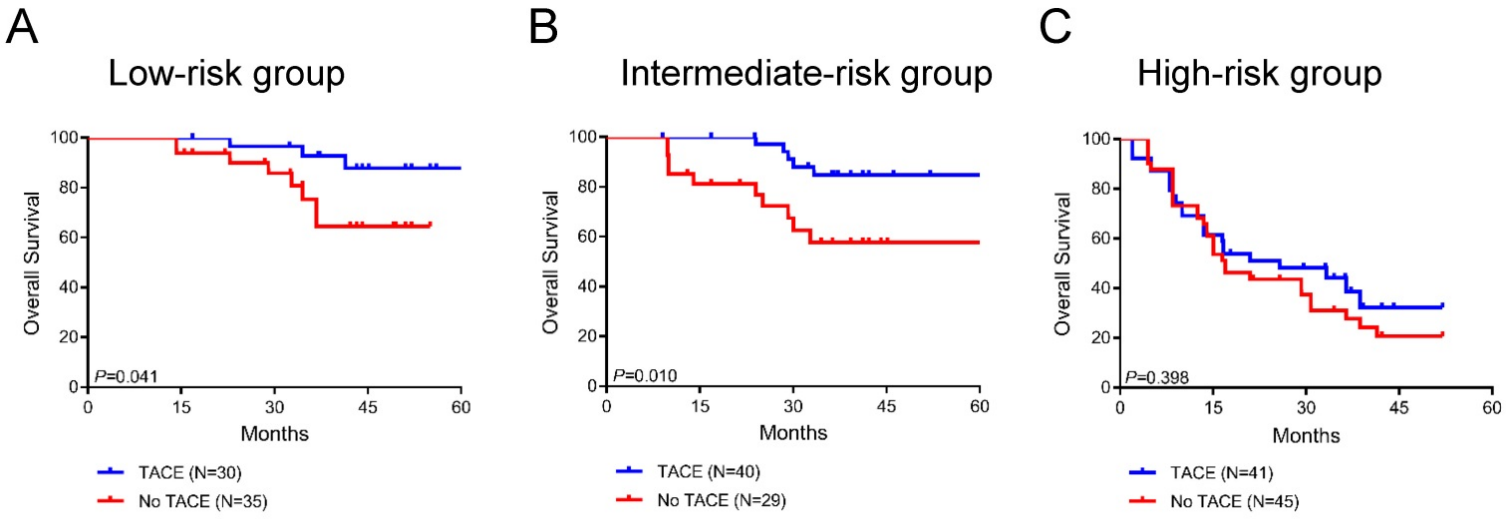
Survival benefit of postoperative TACE in the low-risk (A), intermediate-risk (B) and high-risk (C) groups.

Furthermore, we analyzed the survival benefit from adjuvant TACE in three stratified risk groups. Interestingly, in the low-risk and intermediate-risk groups, postoperative adjuvant TACE significantly prolonged overall survival compared to untreated patients (*P*=0.041 and *P*=0.010, respectively; **Figure 7A-B**). In the low-risk group, the average overall survival times in the TACE and non-TACE groups were 76.6 months (95% CI 70.0-83.2) and 45.9 months (95% CI 40.7-51.2), respectively. In the intermediate-risk group, the average overall survival times in the TACE and non-TACE groups were 61.7 months (95% CI 56.7-66.6) and 58.5 months (95% CI 45.2-71.8), respectively. However, the overall survival of patients in the high-risk group did not show a significant difference between the TACE group and the non-TACE group (*P*=0.398, **Figure 7C**). In addition, adjuvant TACE significantly enhanced OS in the inflamed and immune-excluded groups (*P*=0.023 and *P*=0.003, respectively, **Supplementary Figure 2A-B**) but not in the immune-desert group (*P*=0.771, **Supplementary Figure 2C**). Taken together, these results indicated that the novel immunotype-based risk stratification model could successfully predict adjuvant TACE benefit in HCC patients after surgery.

## Discussion

In the present study, CD8^+^ T cell categories and PD-L1 expression mode were ascertained as two independent predictive factors for prognosis. To precisely predict prognosis in HCC patients, we developed a nomogram incorporating immunotype, PD-L1 expression, BCLC stage and tumor grade. The accurate discrimination of the nomogram was explicated with C-index and calibration curves. This novel stratification model was developed on the basis of total scores calculated by the nomogram and was used to analyze the postoperative overall survival and to evaluate the survival benefit of adjuvant TACE in stratified risk groups.

HCC is a complex, heterogeneous disease with a widely distinct prognosis 30, 31. Currently, several prognostic models for patients with HCC rely on tumor size, tumor number, vascular invasion, differentiation, tumor-node-metastasis (TNM) stage and BCLC stage, which are derived from tumor-cell centered biological phenotypes 1, 32. In fact, accumulative studies have found that various infiltrated immune cells are important prognostic factors for HCC 33-38. Obviously, traditional prognostic models cannot reflect all the biological features of cancer because they neglect the influence of the immunocyte or immune microenvironment. CD8^+^ T cells are the main effector immune cells infiltrating the immune microenvironment. PD-L1 is one of the most important factors inducing T cell exhaustion and immune escape in HCC tumor cells 39. Accumulating evidence indicates that CD8 and PD-L1 can reflect the immune status of the tumor environment, and oncologists tend to use these two indicators to screen candidate patients for PD-L1/PD-1 blockade therapies 26, 40. Concerning the clinical application, we chose CD8 and PD-L1 to reflect the immune status of the tumor microenvironment. The present study characterized CD8^+^ T cell infiltration categories and PD-L1 expression in HCC, which specifically addressed the spatial heterogeneity and function of effective T cells and their prognostic value.

The novel risk stratification model was based on the immunotype, which also included the factors of PD-L1 expression mode, BCLC stage and tumor grade. In comparison with the traditional prognostic model, this novel risk stratification model integrated both immune features and tumor cell-centered biological features, which were reflected in the BCLC stage and tumor grade. Furthermore, risk stratification can visually recognize low-risk, intermediate-risk or high-risk patients by calculating the assigned score of each factor. Additionally, accurate grouping can predict the prognosis more precisely, which is close to the actual survival.

Currently in clinical practice, HCC patients with intermediate and high risks of recurrence (patients who have a single tumor with MVI or major vascular invasion, a single tumor larger than 5 cm, or two or three tumors) in the remnant liver are candidates for enrollment in postoperative adjuvant TACE, but the debate is still ongoing 41, 42. Thus, the identification of predictive biomarkers to be sensitive to postoperative adjuvant TACE is critical, which would prevent excessive toxicities. Accumulated evidence has proven that the immune microenvironment also determines the efficacy of chemotherapy in several tumors 43-48. The persistent immune response targeting tumor cells is essential for successful cytotoxic chemotherapy 49. A previous study indicated that postoperative TACE showed limited prognostic benefit for HCC patients 50. However**,** our novel immunotype-based risk stratification model showed that postoperative TACE discriminatively benefited patients in different risk groups. Patients in the low-risk and intermediate-risk groups benefited from postoperative TACE, while those in the high-risk group did not benefit from TACE. When these results have confirmed the therapeutic effect of adjuvant TACE, especially for low-risk and intermediate-risk patients, more effective therapeutic strategies are needed to benefit high-risk patients. This model emphasizes the role of the immune response (including the PD-L1 expression mode and CD8^+^ T cell infiltration categories) in evaluating the benefit of TACE. Thus, the mechanical interaction between tumor-infiltrated immunocytes and chemotherapy drugs needs to be thoroughly explored. This study demonstrated the value of our novel immunotype-based risk stratification model for predicting the prognosis of survival and benefit from postoperative adjuvant TACE.

However, several limitations must be acknowledged in the present study. This is a single-center, retrospective study, and the sizes of our cohorts are relatively small. The indication of adjuvant TACE is virtually not standard. Although we have recommended postoperative TACE for patients with intermediate and high risks of recurrence, only some patients accepted adjuvant TACE, while the other patients rejected it. We can only assume that various factors, such as socioeconomic status, compliance, and fear, influenced their decision. Therefore, a multicenter prospective study is needed to potently validate this conclusion in the future. In addition, because we selected only CD8^+^ T cells, other crucial immune phenotypes might be neglected. Other types of infiltrated immune cells also play important roles in the immune response, such as tumor-associated macrophages 51-53, tumor-associated neutrophils 52, 54 and myeloid-derived suppressor cell infiltration 54, 55. Further studies should evaluate the role of the whole immunocyte landscape and infiltration heterogenicity.

In conclusion, our data identify that the immune desert, immune exclusion, and inflamed immunotypes and PD-L1 expression are correlated with HCC outcomes and can be used as independent prognostic factors. In addition, we built a novel risk stratification model for predicting postoperative prognosis and adjuvant TACE benefit for HCC patients by integrating this immunotype based on CD8 T cell infiltration, PD-L1 expression and two clinicopathological features. These findings provide a novel predictive tool to predict the prognosis of HCC patients and may improve current predictive systems in terms of counseling patients. Furthermore, immune status can redefine the different risk subgroups of patients with HCC who will benefit from postoperative adjuvant TACE, which could help to develop a more customized method of treatment.

## Supplementary Material

Supplementary figures and tables.

## Figures and Tables

**Table 1:** Baseline clinicopathological characteristics of HCC patients.

Variables	Training cohort (N=105)	Validation cohort (N=115)
Sex		
Male	94 (89.5%)	99 (86.1%)
Female	11 (10.5%)	16 (13.9%)
Age		
<50	33 (31.4%)	47 (40.9%)
≥50	72 (68.6%)	68 (59.1%)
HBV infection		
No	8 (7.6%)	11 (9.6%)
Yes	97 (92.4%)	104 (90.4%)
Cirrhosis		
No	24 (22.9%)	26 (22.6%)
Yes	81 (77.1%)	89 (77.4%)
ALT (U/L)		
<40	48 (45.7%)	51 (44.3%)
≥40	57 (54.3%)	64 (55.7%)
AFP (μg/L)		
<20	35 (33.3%)	41 (35.7%)
≥20	70 (66.7%)	74 (64.3%)
Grade		
G 1-2	59 (56.2%)	84 (73.0%)
G 3-4	46 (43.8%)	31 (27.0%)
Vascular invasion		
No	61 (58.1%)	69 (60.0%)
MVI	34 (32.4%)	34 (29.6%)
Major vascular invasion	10 (9.5%)	12 (10.4%)
Immunotype		
Inflamed	25 (23.8%)	34 (29.6%)
Immune-excluded	33 (31.4%)	36 (31.3%)
Immune-desert	47 (44.8%)	45 (39.1%)
PD-L1 expression		
High	25 (23.8%)	40 (34.8%)
Low	80 (76.2%)	75 (65.2%)
TNM stage		
I	43 (41.0%)	58 (50.4%)
II	35 (33.3%)	26 (22.6%)
III	20 (19.0%)	20 (17.4%)
IV	7 (6.7%)	11 (9.6%)
BCLC stage		
0-A	26 (24.8%)	30 (26.1%)
B	32 (30.5%)	32 (27.8%)
C	47 (44.8%)	53 (46.1%)
TACE		
Yes	53 (50.5%)	58 (50.4%)
No	52 (49.5%)	57 (49.6%)

AFP, alpha fetoprotein; ALT, alanine aminotransferase; BCLC, Barcelona Clinic Liver Cancer; HBV, hepatitis B virus; MVI, microvascular invasion; PD-L1, programmed cell death ligand 1;; TACE, trans-arterial chemoembolization.

**Table 2:** Relationship between clinicopathological features and immunotypes in the training cohort.

Variables	Immunotype			*P*
	Inflamed (N=25)	Immune-excluded (N=33)	Immune-desert (N=47)	
Sex				0.839
Male	22 (88.0%)	29 (87.9%)	43 (91.5%)	
Female	3 (12.0%)	4 (12.1%)	4 (8.5%)	
Age				0.852
<50	9 (36.0%)	10 (30.3%)	14 (29.8%)	
≥50	16 (64.0%)	23 (69.7%)	33 (70.2%)	
HBV infection				0.733
Yes	24 (96.0%)	30 (90.9%)	43 (91.5%)	
No	1 (4.0%)	3 (9.1%)	4 (8.5%)	
Cirrhosis				0.413
No	4 (16.0%)	10 (30.3%)	10 (21.3%)	
Yes	21 (84.0%)	23 (69.7%)	37 (78.7%)	
ALT (U/L)				0.813
<40	10 (40.0%)	16 (48.5%)	22 (46.8%)	
≥40	15 (60.0%)	17 (51.5%)	25 (53.2%)	
AFP (μg/L)				0.712
<20	10 (40.0%)	10 (30.3%)	15 (31.9%)	
≥20	15 (60.0%)	23 (69.7%)	32 (68.1%)	
Grade				0.024
G 1-2	12 (48.0%)	25 (75.8%)	22 (46.8%)	
G 3-4	13 (52.0%)	8 (24.2%)	25 (52.2%)	
Vascular invasion				0.325
No	19 (76.0%)	18 (54.5%)	24 (51.1%)	
MVI	5 (20.0%)	11 (33.3%)	18 (38.3%)	
Major vascular invasion	1 (4.0%)	4 (12.1%)	5 (10.6%)	
TNM stage				0.820
I	11 (44.0%)	15 (45.5%)	17 (45.5%)	
II	11 (44.0%)	8 (24.2%)	16 (44.0%)	
III	3 (12%)	7 (21.2%)	10 (8.0%)	
IV	0 (0%)	3 (9.1%)	4 (4.0%)	
BCLC stage				0.061
0-A	6 (24.0%)	10 (30.3%)	10 (21.3%)	
B	13 (52.0%)	7 (21.2%)	12 (25.5%)	
C	6 (24.0%)	16 (48.5%)	25 (53.2%)	

AFP, alpha fetoprotein; ALT, alanine aminotransferase; BCLC, Barcelona Clinic Liver Cancer; HBV, hepatitis B virus; MVI, microvascular invasion.

**Table 3:** Univariate and multivariate analyses for overall survival in the training cohort.

Variables	Univariable analysis *P*	Multivariable analysis	
		Hazard ratio (95% CI)	*P*
Immunotype	0.001		0.002
Inflamed		Reference	
Immune-excluded		2.365 (1.734-7.616)	0.015
Immune-desert		4.655 (1.600-13.540)	0.008
PD-L1 expression	0.002		0.029
Low		Reference	
High		2.146 (1.067-4.310)	
Sex	0.392		
Male			
Female			
Age	0.540		
<50			
≥50			
HBV infection	0.147		
Yes			
No			
Cirrhosis	0.939		
Yes			
No			
ALT (U/L)	0.631		
<40			
≥40			
AFP (μg/L)	0.141		
<20			
≥20			
Grade	0.001		0.028
G 1-2		Reference	
G 3-4		2.160 (1.085-4.292)	
BCLC stage	<0.001		0.007
0-A		Reference	
B		1.845 (1.104-4.237)	0.032
C		3.846 (1.304-11.363)	0.015

HBV, hepatitis B virus; ALT, alanine aminotransferase; AFP, alpha fetoprotein; BCLC, Barcelona Clinic Liver Cancer.

**Table 4:** Nomogram scores of clinical variables in each subgroup.

Variables	Points
Immunotype	
Inflamed	0
Immune-excluded	56
Immune-desert	100
PD-L1	
Low	0
High	50
Grade	
G 1-2	0
G 3-4	50
BCLC stage	
0-A	0
B	47
C	87
