# Supplementary material for: A Novel Immunotype-based Risk Stratification Model Predicts Postoperative Prognosis and Adjuvant TACE Benefit in Chinese Patients with Hepatocellular Carcinoma
Source: J Cancer. 2021 Mar 15;12(10):2866–76. doi: 10.7150/jca.54408 (PMC8040877; doi:10.7150/jca.54408)
Supplement: Supplementary file 1 — Supplementary figures and tables. [file jcav12p2866s1.pdf]

Supplementary Figures

Supplementary Figure 1

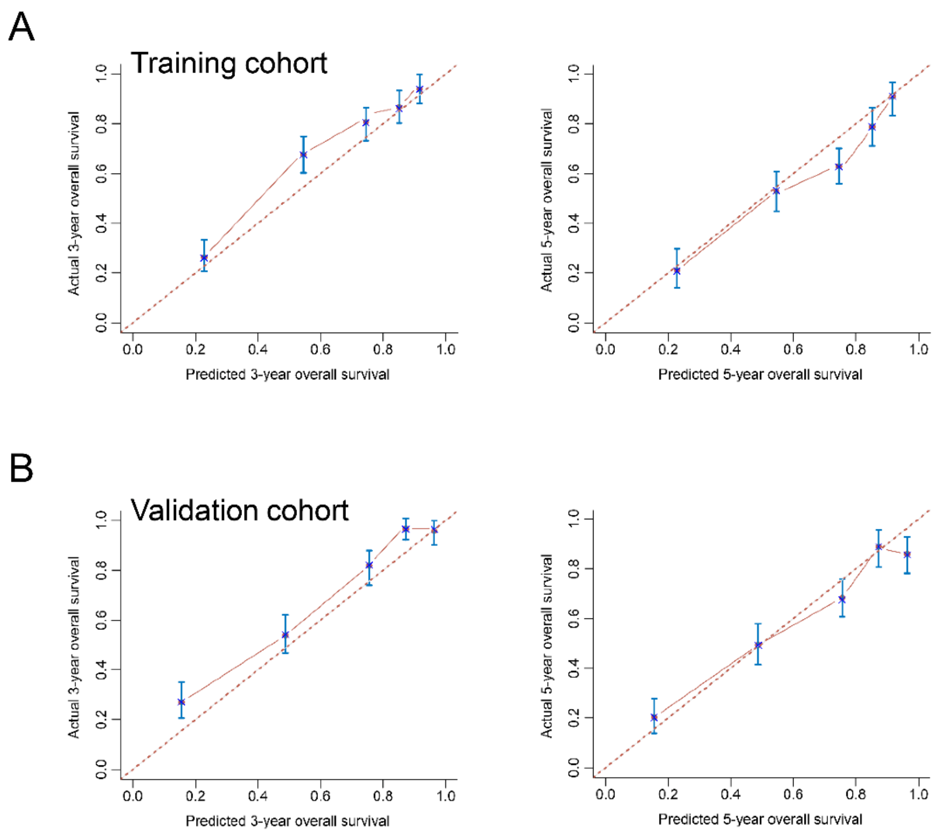

Supplementary Figure 1. Calibration curves for predicting 3- and 5-year overall survival in the training cohort (A), and 3- and 5-year (D) overall survival in the validation cohort (B).

Supplementary Figure 2

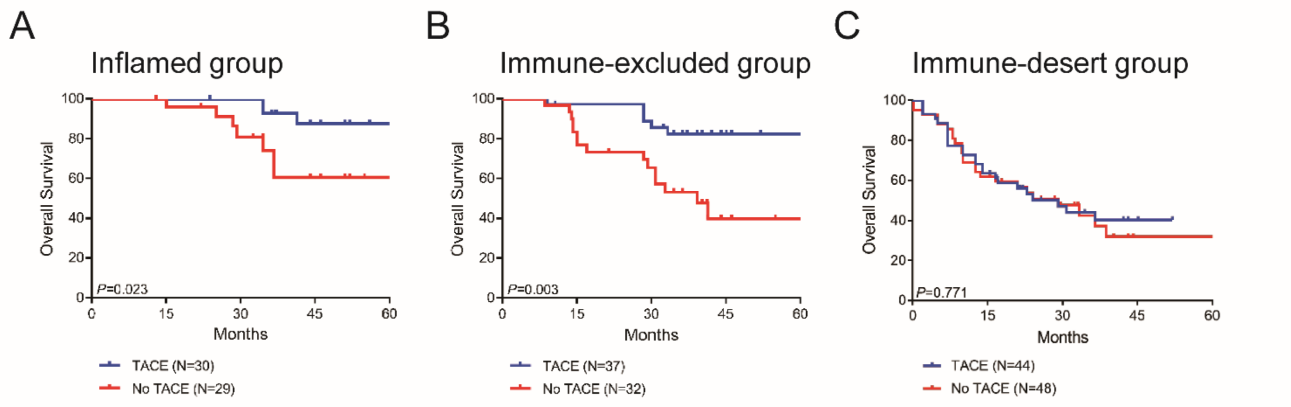

Supplementary Figure 2. Survival benefit of postoperative TACE in the inflamed (A), immune-excluded (B) and immune-desert (C) groups.

Supplementary Table 1. Univariate and multivariate analyses for recurrence-free survival in the training cohort.

Variables	Univariable analysis *P*	Multivariable analysis	
		Hazard ratio (95% CI)	*P*
Immunotype	0.038		0.015
Inflamed		Reference	
Immune-excluded		3.313 (1.133-11.757)	0.044
Immune-desert		4.330 (1.324-14.159)	0.017
Sex	0.682		
Male			
Female			
Age	0.458		
<50			
≥50			
HBV infection	0.107		
Yes			
No			
Cirrhosis	0.474		
Yes			
No			
ALT (U/L)	0.288		
<40			
≥40			
AFP (μg/L)	0.580		
<20			
≥20			
Grade	<0.001		0.001
G 1-2		Reference	
G 3-4		4.566 (1.832-11.364)	
BCLC stage	0.001		<0.001
0-A		Reference	
B		5.051 (1.621-15.625)	0.005
C		7.692 (1.761-33.333)	0.007
PD-L1 expression	<0.001		<0.001
Low		Reference	
High		6.667 (2.660-16.667)	

HBV, hepatitis B virus; ALT, alanine aminotransferase; AFP, alpha fetoprotein.
